# An Account of a Painful Swelling of the Perinæum, Which Took Place Immediately after Delivery, and Terminated in Sphacelus

**Published:** 1788

**Authors:** Thomas Reeve

**Affiliations:** Surgeon at Boterdale in Suffolk


II. *An Account of a painful Swelling of the Perinæum, which took place immediately after delivery, and terminated in Sphacelus. Communicated in a Letter to Dr.* Simmons *by Mr.* Thomas Reeve, *Surgeon at Boterdale in Suffolk.*

ON the 13th of September, 1787, I was ſent for, in the afternoon, to a female patient about thirty-two years of age, who had been that morning taken in labour of her firſt child. Till ten o'clock in the evening the pains were ſlight, with conſiderable intervals between them, but they then increaſed both in ſtrength and frequency. The head was now low down in the pelvis, and the child gradually advancing, when, in the laſt pain but one, the patient complained of an odd ſenſation, unconnected with labour pains, which produced hyſteric ſymptoms, that continued to diſtreſs her a conſiderable time. The next pain brought forth the child without any aſſiſtance, and about five minutes afterwards, the placenta was expelled without difficulty.

Hitherto nothing very alarming had made its appearance, but now ſhe was attacked with excruciating pain *in perinæo*, different from what ſhe

ſhe had ever deſcribed during labour, as it was much more acute ; and it continued to increaſe till a ſyncope came on. As ſoon as ſhe recovered, ſhe complained of a hard tumour in the perinæum; which tumour, in a very ſhort time, ſpread ſo rapidly, that it occupied the whole of the labium pudendi ſiniſtrum, and extended to the opening in Poupart's ligament.

In the morning of the 14th, finding the ſwelling very tenſe, with the integuments ready to burſt, I applied to it the vegeto-mineral water on compreſſes of ſoft linen for the firſt twelve hours, hoping that cold applications would produce a reabſorption of the extravaſated fluids, and diſcuſs the ſwelling, which now was equal in magnitude to the body of a full grown fœtus.

On the 15th, the tumour appeared much diſcoloured, with a livor on many parts of it; and, on the 16th, notwithſtanding every effort to obviate it, a complete ſphacelus ſeized the whole of the ſwollen parts: and now ſymptoms of irritation, uſually attendant on gangrene, ran ſo high, that her life ſeemed to be in very imminent danger. The bark was given immediately in ſubſtance, in as large doſes, and as often repeated, as the ſtomach could bear. Warm antiſeptic fomentations and poultices were applied to the parts three or four times in a day, and the room kept well ventilated, as the ſmell from the ſore was almoſt intolerable.

On the 18th, a ſeparation of the dead from the living parts began to take place, with a copious diſcharge of pus and coagulated blood. From this time ſhe continued gradually to recover; and nature, aſſiſted by tonics and a nutritious diet, produced a regeneration of the parts deſtroyed, ſo that in three weeks ſhe was perfectly well.

From the commencement of the ſwelling, ſhe laboured under a ſuppreſſion of urine, which obliged me to introduce the catheter whenever an over-diſtention of the bladder produced pain and uneaſineſs.

*Query.* Would punctures, made into the tumour with the point of a lancet, have prevented that deſtruction of the ſoft parts conſequent to the putrefaction of the extravaſated blood?

*Boterdale,*
April 22, 1788.

